# Mesenchymal stem cell repression of Th17 cells is triggered by mitochondrial transfer

**DOI:** 10.1186/s13287-019-1307-9

**Published:** 2019-08-01

**Authors:** Patricia Luz-Crawford, Javier Hernandez, Farida Djouad, Noymar Luque-Campos, Andres Caicedo, Séverine Carrère-Kremer, Jean-Marc Brondello, Marie-Luce Vignais, Jérôme Pène, Christian Jorgensen

**Affiliations:** 1 Laboratorio de Inmunología Celular y Molecular, Centro de Investigación Biomédica, Facultad de Medicina, Universidad de los Andes, Santiago, Chile; 2 IRMB, Univ Montpellier, INSERM, Hôpital Saint-Eloi, 80 avenue Augustin Fliche, 34295 Montpellier CEDEX 5, France; 3 CHU Montpellier, F-34295 Montpellier, France; 4 Universidad San Francisco de Quito, Hospital de los Valles, Quito, Ecuador

**Keywords:** Mitochondria transfer, Mesenchymal stem cells, T cell, Th17, Immunomodulation

## Abstract

**Background:**

Mesenchymal stem cells (MSCs) are multipotent cells with broad immunosuppressive capacities. Recently, it has been reported that MSCs can transfer mitochondria to various cell types, including fibroblast, cancer, and endothelial cells. It has been suggested that mitochondrial transfer is associated with a physiological response to cues released by damaged cells to restore and regenerate damaged tissue. However, the role of mitochondrial transfer to immune competent cells has been poorly investigated.

**Methods and results:**

Here, we analyzed the capacity of MSCs from the bone marrow (BM) of healthy donors (BM-MSCs) to transfer mitochondria to primary CD4^+^CCR6^+^CD45RO^+^ T helper 17 (Th17) cells by confocal microscopy and fluorescent-activated cell sorting (FACS). We then evaluated the Th17 cell inflammatory phenotype and bioenergetics at 4 h and 24 h of co-culture with BM-MSCs. We found that Th17 cells can take up mitochondria from BM-MSCs already after 4 h of co-culture. Moreover, IL-17 production by Th17 cells co-cultured with BM-MSCs was significantly impaired in a contact-dependent manner. This inhibition was associated with oxygen consumption increase by Th17 cells and interconversion into T regulatory cells. Finally, by co-culturing human synovial MSCs (sMSCs) from patients with rheumatoid arthritis (RA) with Th17 cells, we found that compared with healthy BM-MSCs, mitochondrial transfer to Th17 cells was impaired in RA-sMSCs. Moreover, artificial mitochondrial transfer also significantly reduced IL-17 production by Th17 cells.

**Conclusions:**

The present study brings some insights into a novel mechanism of T cell function regulation through mitochondrial transfer from stromal stem cells. The reduced mitochondrial transfer by RA-sMSCs might contribute to the persistence of chronic inflammation in RA synovitis.

**Electronic supplementary material:**

The online version of this article (10.1186/s13287-019-1307-9) contains supplementary material, which is available to authorized users.

## Background

Mesenchymal stem cells (MSCs) are multipotent and immunoregulatory stem cells that limit the progression and severity of autoimmune diseases [1–3]. MSC immunosuppressive functions are mediated through cell–cell contacts and the secretion of soluble factors, particularly inducible nitric oxide synthase by murine MSCs and indoleamine-2,3-dioxygenase by human MSCs [4], as well as IL-6, TGF-β1 and PGE2, IL-1RA, and HLAG5s [5–8]. We and others demonstrated that MSCs prevent T cell differentiation into T helper (Th)17 and Th1 cells and induce T regulatory (Treg) cells in vitro [9–11]. This MSC inhibitory effect requires cell–cell contacts through ICAM-1 and the release of soluble factors, such as PGE2 [9, 11]. MSC immunomodulatory properties are not intrinsic, but are induced by inflammatory cytokines [9, 12–14]. Thus, MSCs establish direct interactions with T cells in physiological conditions where MSCs play a key role in T cell homeostasis and also in pathological conditions where infused MSCs have immunoregulatory properties.

It has been shown that MSCs make tunneling nanotube (TNT) connections with various cell types, leading to intercellular exchange of mitochondria [15, 16]. We previously demonstrated that the dynamic transfer of mitochondria to carcinoma cells induces changes in malignant cell proliferation and glucose uptake [17]. Moreover, mitochondrial transfer via the TNT connections between MSCs and non-malignant cells with mitochondrial damage allows rescuing the mitochondrial function in the receiving cells [16, 18]. TNT-mediated transfer of MSC mitochondria to cancer cells increases their metabolic activity and resistance to therapy. It has been proposed that such transfer is regulated by the mitochondrial Rho-GTPase Miro that favors mitochondrial trafficking along cytoskeleton fibers [15, 16, 18]. In vivo, the transfer of MSC mitochondria to damaged bronchial epithelial cells modifies the metabolism and restores the functionality of the receiving cells, thus limiting cell damage and apoptosis [19, 20]. Thus, intercellular mitochondrial transport modifies the recipient cell bioenergetics and stimulates their proliferation and functions. Concerning immune cells, it has been shown that MSCs extensively transfer mitochondria to macrophages, partially through TNT, thus increasing their phagocytic activity. However, it is not known whether MSCs can transfer mitochondria to pathogenic Th17 cells and the consequence of this event, although it is widely acknowledged that MSCs regulate the balance between pathogenic Th17 cells and Treg cells to improve autoimmune disease outcomes.

In this study, we investigated the role of mitochondrial transfer in the MSC-mediated modulation of Th17 cell function. To this aim, we co-cultured memory Th17 cells and bone marrow (BM)-MSCs to investigate MSC capacity to transfer mitochondria to Th17 cells and the effect of this transfer on Th17 cell phenotype, function, and metabolism. Then, using an artificial method to transfer mitochondria (i.e., MitoCeption), we assessed the direct effect of BM-MSC mitochondrial transfer on the Th17 cell phenotype. Finally, to determine the influence of the inflammatory environment in the joints of patients with rheumatoid arthritis (RA) on mitochondrial transfer from MSCs to T cells, we compared mitochondrial transfer from RA synoviocytes and healthy BM-MSCs to CD4^+^ T cells. Altogether, our results highlight that mitochondrial transfer from MSCs is a novel immunoregulatory mechanism for inhibiting Th17 cells that could be impaired in RA synoviocytes.

## Methods

### Isolation and characterization of human MSCs

Human MSCs, isolated from the BM of three healthy donors after written consent, were obtained from the Établissement Français du Sang (EFS; Grenoble, France) and used at the third or fourth passage. MSCs were cultured in α-MEM medium (Lonza, Switzerland) supplemented with 10% fetal calf serum (FCS) (Sigma, USA), 2 mM glutamine, 100 U/ml penicillin, 100 mg/ml streptomycin (without antibiotics for PCR assays with mitochondrial DNA and for T cell bioenergetics analysis), and 1 ng/ml basic FGF (Bio-Techne, UK). To simulate an inflammatory environment, MSCs were pre-incubated with TNF-α (10 ng/ml) and IFN-γ (20 ng/ml) (R&D, France) for 48 h.

### Isolation and culture of human T cells

Polyclonal CD4^+^ T cell populations were prepared from peripheral blood mononuclear cells (PBMCs) from five healthy donors (EFS, Montpellier, France) after written consent. CD4^+^ T cell isolation, stimulation, and culture protocols were as previously described [9]. Briefly, in vivo polarized CD4^+^ Th17 cells were sorted by flow cytometry based on the fact that the chemokine receptor CCR6 is expressed almost exclusively by the Th17 cell subset and not by CD4^+^ Th1 and Th2 cells. CD4^+^CCR6^+^ and CD4^+^CCR6^−^ T cells were propagated by weekly stimulation with an irradiated feeder cell mixture (PBMCs and JY cell line) with PHA (Lonza, France) and recombinant IL-2 (2 ng/ml; Bio-Techne, UK). To prevent any possible derivation of the T cell lines due to culture conditions, cells were stimulated at most thrice, and their production of IL-17 and expression of RoRγt were regularly tested. T cells were used 10 to 15 days after the latest propagation phase, a time window that corresponds to T cells in a resting phase. All culture procedures were carried out in Yssel’s medium, supplemented with 2% human AB^+^ serum (EFS, Lyon, France).

### Isolation and characterization of human RA synovial stromal stem cells (RA-sMSCs)

The use of clinical biopsies and blood samples from patients with RA was approved by the ethics committee of Montpellier University Hospitals (DC-2008-417—coordinator C Jorgensen). Culture conditions were as for BM-MSCs, and isolation was performed as previously described [21].

### Co-culture experiments

MSCs were labeled with the MitoTracker Green FM (M7514), MitoTracker Red CMXRos, or MitoTracker Deep Red FM (far red) fluorescent mitochondrial dyes (Molecular Probes, France) according to the manufacturer’s instructions, 24 h before co-culture. In some experiments, T cells were labeled with the CellTracker Green fluorescent dye (Molecular Probes, France) according to the manufacturer’s instructions. After labeling, MSCs were harvested, extensively washed in PBS, and co-cultured with Th17 cells (10^6^ cells, MSC to T cell ratio 1:25) in 24-well culture plates (BD, Germany) activated with anti-CD3 and anti-CD28 monoclonal antibody (mAb)-coated beads (Expander Beads, ThermoFisher, UK) in the presence of 30 U/ml of IL-2 for 3 days. As a control, T cells were cultured without MSCs. At two time points during co-culture (4 h and 24 h), non-adherent T cells were harvested, and MitoTracker dye uptake (as a sign of mitochondrial transfer from MSCs) was analyzed by flow cytometry.

### Flow cytometry

Flow cytometry analysis of T cells and MSCs was carried out as previously described [9] using PE-conjugated anti-CCR6 (Bio-Techne, UK), Fluo-conjugated anti-CD3 and anti-CD4 mAbs, and isotype control (BD, Germany) and a FACSCanto or FACSFortesa flow cytometer. Data (10,000 events/sample) were collected using the DIVA software (BD, Germany). Cell sorting was performed on a FACSAria® flow cytometer (BD, Germany).

Intracellular staining of T cells was performed after 4 h of activation with PMA (20 ng/ml; Merck, Germany) and ionomycin (1 mg/ml; Merck, Germany), in the presence of brefeldin A (10 mg/ml; Sigma, USA). When necessary, cells were stained with PE-conjugated anti-CCR6 (Bio-Techne, UK), Fluo-conjugated anti-CD3 and anti-CD4 mAbs, and isotype control (BD, Germany). Then, cells were fixed with Cytofix/Cytoperm buffer (ThermoFisher, UK) at 4 °C overnight and stained with fluorochrome-conjugated mAbs diluted in Perm/Wash buffer (ThermoFisher, UK), according to the manufacturer’s specifications. Cytokine production was assessed by flow cytometry using anti-IL-17–Percp5.5, anti-IL-CD25–APC, anti-IL-10–PECY7, and anti-IFN-γ–APCCY7 (ThermoFisher, UK) mAbs. FOXP3 expression was assessed with an anti-FOXP3 PE-conjugated mAb (ThermoFisher, UK). All mAbs were used at the optimal concentrations previously determined in independent experiments on the basis of the manufacturer’s recommendations.

T cell proliferation was assessed with the CellTrace Violet Cell Proliferation Kit for flow cytometry (Molecular Probes, France) according to the manufacturer’s instructions.

### PCR assays with mitochondrial DNA

MSC mitochondrial DNA (mtDNA) in T cells was quantified by PCR, based on the distinct haplotypes of the MSC and T cell donors. To increase the assay specificity, a supplementary modification was introduced at position n-2 relative to the SNP site and at the 3′ end of the PCR primers. MSC mtDNA from one donor was amplified with the primers 5′-TTAACTCCACCATTAGCACC-3′ [22] and 5′-AGTATTTATGGTACCGTCCG-3′ (positions 15971–16143) and MSC mtDNA from the two other donors with the primers 5′-TAACAGTACATAGCACATACAA-3′ and 5′-GAGGATGGTGGTCAAGGGA-3′ (universal sequencing primer [22]) (positions 16298–16410).

### Confocal microscopy

Cells were seeded on culture dishes with glass bottom, and images of live cells were taken with a Carl Zeiss LSM 5 LIVE (LSM 510 META and LSM 5 LIVE) DuoScan Laser Scanning microscope immediately after 4 h of MSC-T cell co-culture. 3D reconstruction was done using the Imaris Bitplane software.

### T cell bioenergetics

Oxygen consumption rate (OCR) was measured using the XF24 analyzer (Seahorse Biosciences, North Billerica, MA, USA). Briefly, XF24 24-well plates were coated with Cell-Tak (BD Biosciences, France), as described in the Seahorse protocol. T cells (10^6^ cells/well) were plated in unbuffered XF assay medium–modified DMEM (Seahorse Biosciences) at pH 7.4, supplemented with 10 mM glucose, 2 mM glutamate, and 1 mM sodium pyruvate, and incubated at 37 °C without CO_2_. OCR was measured (4 wells per condition) in basal conditions and after sequential addition of oligomycin (1 μM), carbonylcyanide-4-trifluoromethoxyphenylhydrazone (FCCP) (1 μM), rotenone (100 nM), and antimycin A (1 μM). Three readings were taken after each addition. Instrument background was measured in separate control wells using the same conditions but without cells. T cells from three independent co-cultures were used for these experiments.

### Isolation of MSC mitochondria and transfer to T cells (MitoCeption)

Mitochondria were isolated using the Mitochondria Isolation Kit for Cultured Cells (ThermoScientific) following the manufacturer’s instructions. At least 1 × 10^6^ MitoTracker-stained MSCs were used to obtain between 50 and 100 μg of mitochondria. To reduce contamination from cytosolic elements, a final centrifugation was performed at 3000*g* for 15 min. Isolated mitochondria were resuspended in Yssel’s medium, supplemented with 2% human AB^+^ serum (EFS, Lyon, France), and maintained on ice for immediate transfer into CD4^+^CD45^+^CCR6^+^ T cells. To this aim, 10 μg of mitochondria was used for 2.5 × 10^5^ T cells, as previously described [17]. Then, culture plates were centrifuged at 1500*g* at 4 °C for 15 min and incubated at 37 °C, 5%CO_2_, for 24 h. The following day MitoCeption efficiency was verified by flow cytometry analysis of the MitoTracker signal in T cells (Additional file 1: Figure S1).

### Statistical analyses

Data were analyzed using GraphPad Prism 6 (GraphPad Software Inc., San Diego, CA). The non-parametric Friedman test was used to evaluate statistical differences among paired multiple samples, and the non-parametric Mann–Whitney test to compare two variables. Differences were considered statistically significant when *P* < 0.05 (*P < 0.05, **P < 0.01, ***P < 0.001). All data are presented as mean values ± SEM.

## Results

### Uptake of mitochondria by primary human CD4^+^CCR6^+^CD45R0^+^ T cells co-cultured with BM-MSCs

**Fig. 1:**
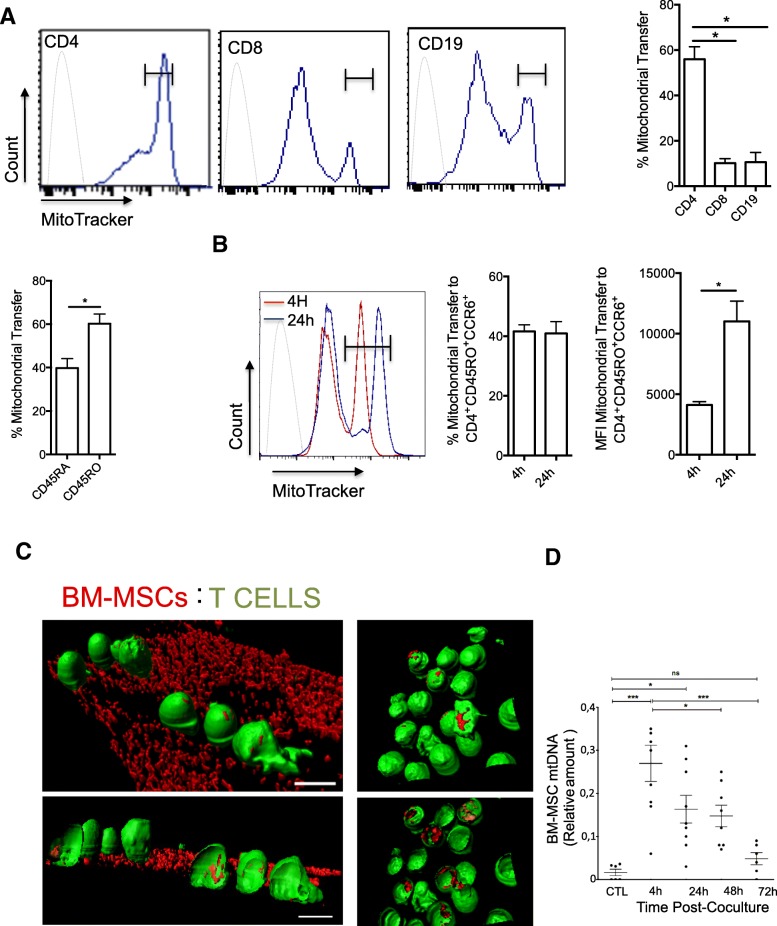
Primary human Th17 cells take up mitochondria after co-culture with BM-MSCs. **a** MitoTracker-labeled BM-MSCs were co-cultured with PBMCs for 4 h, and then mitochondrial transfer to naive CD4^+^ T cells, CD8^+^ T cells, and B cells was evaluated by flow cytometry. **b** Sorted Th17 cells were co-cultured with MitoTracker-labeled BM-MSCs for 4 and 24 h, and mitochondrial transfer was assessed by flow cytometry analysis. Mitochondrial transfer is expressed as the percentage of MitoTracker-positive T cells relative to all T cells and as the mean fluorescence intensity (MFI). **c** 3D reconstruction of confocal microscopy images of BM-MSC mitochondrial transfer to T cells after co-culture for 4 h (MSC to T cell ratio = 1:25). BM-MSCs and T cells were labeled with MitoTracker Red CMXRos and CellTracker Green, respectively, before co-culture. Confocal microscopy images obtained immediately after co-culture show T cells attached to BM-MSCs (left panels) and non-adherent T cells (right panels). Scale bar 10 μm. **d** Detection of BM-MSC mitochondrial DNA (mtDNA) by qPCR in T cells before c-culture (CTL), after 4 h of co-culture, and then at 24, 48, and 72 h. Data are the mean ± SEM of at least 3 independent experiments using T cells from five different PBMC donors and BM-MSCs from three donors

To determine whether mitochondria are transferred from BM-MSCs into primary lymphocytes, BM-MSCs were labeled with the MitoTracker Green probe and co-cultured with human PBMCs from healthy donors. After 4 h of co-culture, PBMCs were harvested and labeled with fluorochrome-conjugated mAbs to identify CD4^+^, CD8^+^, and CD19^+^ T cells. FACS analysis showed that mitochondria were transferred from BM-MSCs into all studied lymphocyte subsets, particularly in CD4^+^ T cells (56% in CD4^+^ vs 17% in CD8^+^ T cells and 24% in B cells) (Fig. 1a). Moreover, mitochondria uptake was higher in CD4^+^ memory T cells (CD45RO^+^) than that in naive CD4^+^ T cells (CD45RA^+^), and 60% of memory T cells were CCR6^+^ cells (Th17 memory phenotype) (Fig. 1a). As memory CD4^+^ T cells, and particularly those expressing CCR6, were the best recipients of BM-MSC mitochondria, the subsequent analyses focused on the impact of mitochondrial transfer to pro-inflammatory Th17 cells. To this aim, FACS-sorted CD4^+^CCR6^+^CD45RO^+^ T cells from PBMCs of five healthy donors were stimulated and amplified in vitro to obtain an enriched Th17 population. Then, day 10 resting Th17 cells were co-cultured with MitoTracker-labeled BM-MSCs isolated from three healthy donors for 4 h and 24 h. FACS analysis of non-adherent T cells showed that BM-MSCs could transfer mitochondria to Th17 cells after 4 and 24 h of co-culture. Of note, although the percentage of mitochondrial transfer did not change between 4 and 24 h, the mean fluorescence intensity (MFI) was higher after 24 h of co-culture, suggesting that only a fraction of Th17 cells could receive mitochondria from BM-MSCs (Fig. 1b). Mitochondrial transfer was completely abolished by mechanical shaking of cells (data not shown), indicating that it occurred through a contact-dependent mechanism. Moreover, incubation of T cells with culture medium conditioned by MitoTracker-labeled BM-MSCs, but without BM-MSCs, did not lead to a fluorescence signal increase in T cells (data not shown), ruling out the possibility of passive staining of T cell mitochondria due to MitoTracker probe leak. To determine whether BM-MSC mitochondria enter Th17 cells, CellTracker-labeled T cells (green) were analyzed by confocal microscopy after co-culture (4 h) with MitoTracker Red CMXRos-labeled BM-MSCs (Fig. 1c). MSC mitochondria (red) were observed in some T cells attached to the BM-MSCs (Fig. 1c) and also in some T cells in suspension (Fig. 1c, right panel).

To confirm the transfer of MSC mitochondria in T cells during co-culture, MSC mtDNA was quantified in T cells (Fig. 1d) by taking advantage of the different mtDNA haplotypes of BM-MSCs and T cells (different donors for each cell type). First, mtDNA was isolated from MSCs (three donors) and T cells (one donor), and their variable D-loop regions were sequenced. This allowed the detection of specific SNPs, and the design of PCR primers to specifically target and amplify MSC (and not T cell) mtDNA. After 4 h of co-culture (MSC to T cell ratio 1:25), non-adherent T cells were harvested and cultured with IL-2 and without BM-MSCs for up to 72 h. PCR analysis of MSC mtDNA in T cells after the 4 h of co-culture and then at 24, 48, and 72 h of incubation with IL-2 showed that MSC mtDNA could be detected in T cells after 4 h of co-culture and up to 48 h with IL-2, but not at the 72-h time point.

Altogether, these results indicate that mitochondria are efficiently transferred from human BM-MSCs into Th17 cells via cell-to-cell interactions already after 4 h of co-culture.

### Co-culture with BM-MSCs leads to a reduction of Th17 cell responses and Treg cell generation

**Fig. 2:**
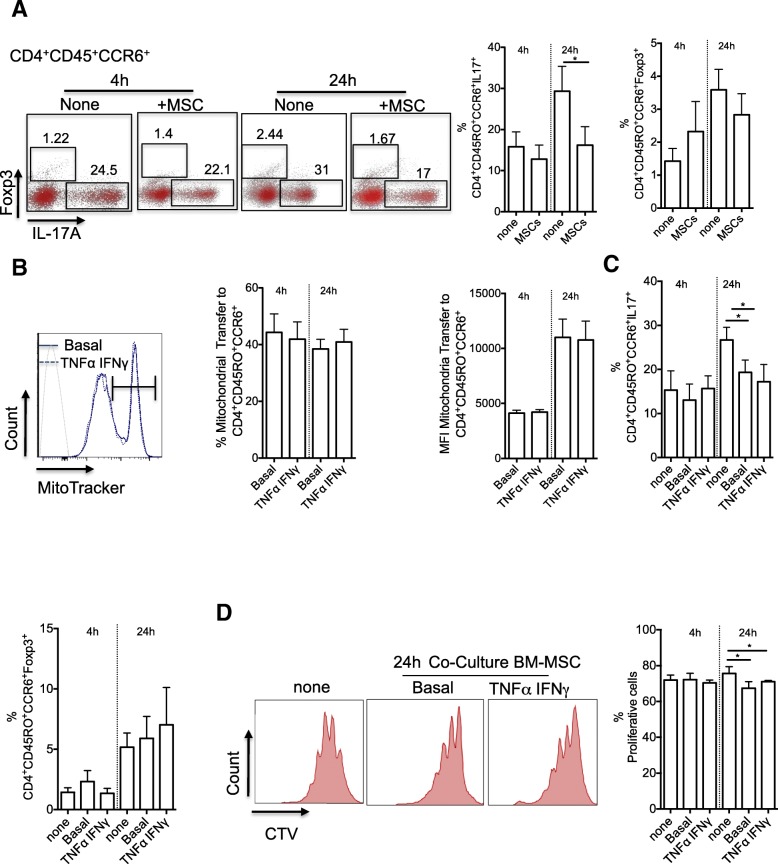
Mitochondrial transfer from BM-MSCs significantly reduces IL-17 production independently of TNF-α/IFN-γ stimulation. **a** Representative dot plots of FOXP3 and IL-17 expression (left panels) in Th17 cells after 3 days of activation by incubation with beads coupled to anti-CD3/CD28 antibodies following (+) or not (−) co-culture with BM-MSCs for 4 or 24 h. Graphs (right panels) show the quantification of the results. **b** BM-MSC mitochondrial transfer to Th17 cells after 4 and 24 h of co-culture. BM-MSCs were pre-incubated or not (Basal) with TNF-α and IFN-γ. Mitochondrial transfer is expressed as the percentage of MitoTracker-positive T cells relative to all T cells and as the mean fluorescence intensity (MFI). **c** IL-17 and FOXP3 expression quantification in Th17 cells co-cultured or not (None) with BM-MSCs pre-incubated or not (Basal) with TNF-α and IFN-γ. **d** Proliferation of Th17 cells after 3 days of activation by incubation with beads coupled to anti-CD3/CD28 antibodies following co-culture or not (None) with BM-MSCs pre-activated or not (Basal) with TNF-α and IFN-γ. Data are the mean ± SEM of at least 3 independent experiments using PBMCs from five donors and BM-MSCs from three donors (MSC to T cell ratio = 1:25)

**Fig. 3:**
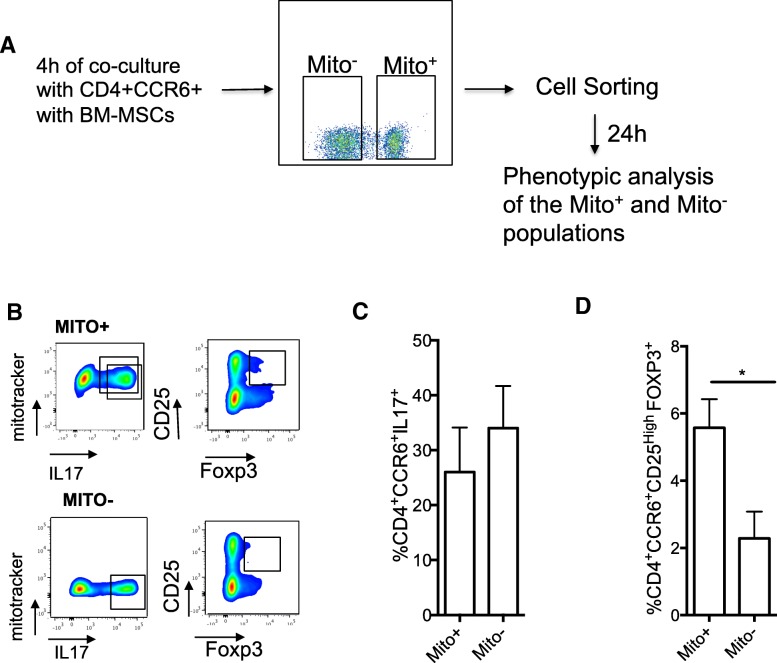
Mitochondrial transfer from BM-MSCs significantly increases the generation of Treg cells. **a** Schematic representation of the cell sorting experiment. Th17 cells were co-cultured with MitoTracker (Mito)-labeled BM-MSCs for 4 h, and then Mito^+^ and Mito^−^ Th17 cells were isolated by cell sorting. **b** Representative dot plots of cells with the Th17 and Treg phenotypes. **c** IL-17 expression quantification 24 h after cell sorting of Mito^+^ and Mito^−^ Th17 cells by flow cytometry. **d** CD25 and FOXP3 expression quantification 24 h after sorting of Mito^+^ and Mito^−^ cells by flow cytometry. Data are the mean ± SEM of 3 independent experiments using PBMCs from four donors and BM-MSCs from three donors (MSC to T cell ratio = 1:25)

Our previous study showed that after long-term co-culture, MSCs interfere with the functional activity of Th17 cells, a CD4^+^ T cell population that plays an important role in the development of autoimmune diseases, such as RA [9, 11]. To investigate whether short-term (4 or 24 h) co-culture also could alter Th17 cell function and induce the expression of Treg markers, particularly FOXP3, CD4^+^CCR6^+^CD45RO^+^ T cells sorted from PBMCs of five healthy donors were stimulated and amplified in vitro to obtain a T cell population enriched in Th17 cells. Then, resting Th17 cells were co-cultured with BM-MSCs isolated from three healthy donors for 4 h, followed by harvesting of non-adherent Th17 cells and stimulation with anti-CD3 and anti-CD28 antibody-coated beads for 3 more days. Finally, FACS-based quantification of IL-17 and FOXP3 expression showed that compared with not co-cultured Th17 cells, production of IL-17 was reduced only after co-culture with BM-MSCs for 24 h (Fig. 2a). Conversely, the FOXP3 expression level was not significantly affected by co-culture with BM-MSCs (Fig. 2a). Then, to mimic a pro-inflammatory environment, BM-MSCs were pre-incubated with TNF-α (10 ng/ml) and INF-γ (20 ng/ml) for 48 h before co-culture. This pre-incubation step did not affect (1) mitochondrial transfer from BM-MSCs to Th17 cells (Fig. 2b), (2) BM-MSC suppressive effect on Th17 cell phenotype and function (Fig. 2c), and (3) BM-MSC inhibitory effect on Th17 cell proliferation (Fig. 2d).

Then, to monitor the fate of Th17 cells that acquired or not mitochondria from BM-MSCs, Th17 cells were co-cultured with BM-MSCs for 4 h, and Th17 cells that received mitochondria (Mito^+^) or not (Mito^−^) were separated by cell sorting (Fig. 3a). FACS analysis performed 24 h after cell sorting showed that the FOXP3 signal was higher in Mito^+^ than in Mito^−^ T cells (Fig. 3b). Moreover, Mito^+^ T cells produced less IL-17 than Mito^−^ T cells, although the difference was not significant (Fig 3c). Finally, the percentage of CD4^+^CCR6^+^CD25^High^FOXP3^+^ cells (i.e., Th17 cells with a regulatory phenotype) was higher among Mito^+^ than Mito^−^ cells. Altogether, these data suggest that mitochondrial transfer from BM-MSCs to Th17 cells results in a reduced production of pro-inflammatory cytokines and induces a Treg phenotype in effector memory Th17 cells.

### Effect of BM-MSC mitochondrial transfer on the energetic metabolism of recipient Th17 cells

**Fig. 4:**
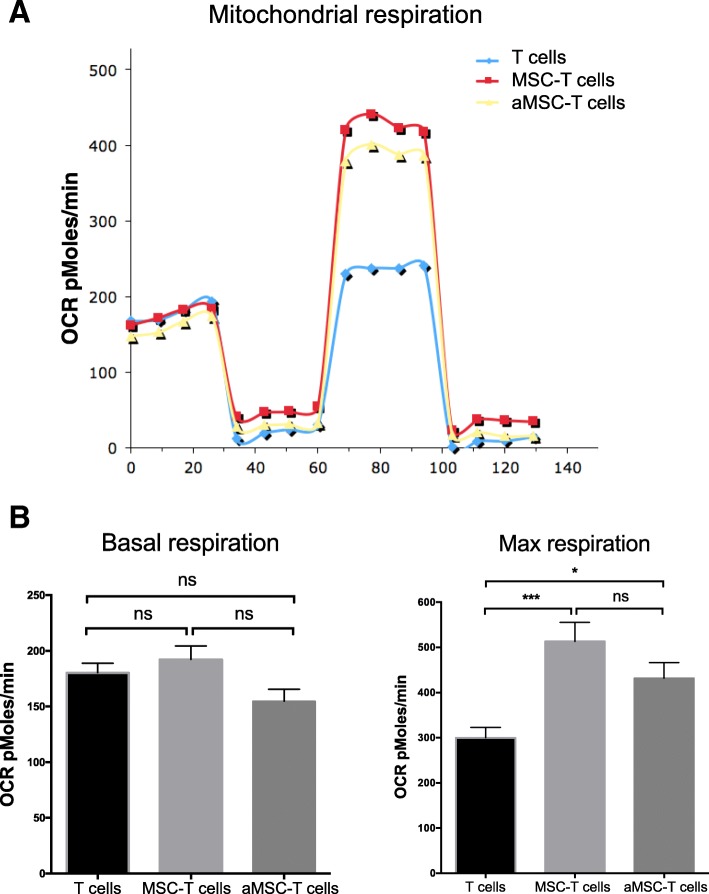
Effects of BM-MSC mitochondria on T cell OXPHOS. After co-culture (4 h) with BM-MSCs pre-activated (aMSC) or not with TNF-α/IFN-γ, T cells were cultured for 48 h and then their oxygen consumption rates (OCRs) were measured (Seahorse system). **a** Results of a representative experiment at 48 h after co-culture. The T cell OCR values were determined in basal conditions and after consecutive addition of oligomycin, FCCP, and rotenone/antimycin A. **b** OCR quantification (basal condition) in Th17 cells co-cultured or not with BM-MSCs pre-activated (aMSC) or not (MSC) with TNF-α and IFN-γ. **c** OCR quantification to evaluate the maximal respiration in Th17 cells co-cultured or not with BM-MSCs pre-activated (aMSC) or not (MSC) with TNF-α and IFN-γ. Data are the mean values ± SEM of 4 independent experiments using BM-MSCs from three healthy donors (MSC to T cell ratio = 1:25)

Mitochondria are crucial for the cell energetic metabolism and oxidative phosphorylation (OXPHOS). To determine the effect of BM-MSC-T cell co-culture on T cell metabolism, non-adherent T cells were collected after 4 h of co-culture with BM-MSCs and cultured in the presence of IL-2 for 24 or 48 h. OXPHOS quantification using the Seahorse extracellular flux technology showed that co-culture had no effect on the T cell basal respiration levels (OCR) compared with control (T cells alone) (Fig. 4a). Conversely, the maximal T cell respiration was increased (as observed upon FCCP addition) in co-cultured T cells. Similar results were obtained using BM-MSCs from three different donors (Fig. 4b for basal respiration-level data). The maximal respiration was increased by 1.7-fold in T cells co-cultured for 4 h (Fig. 4c).

### Artificial transfer of BM-MSC mitochondria to T cells alters Th17 cell function

**Fig. 5:**
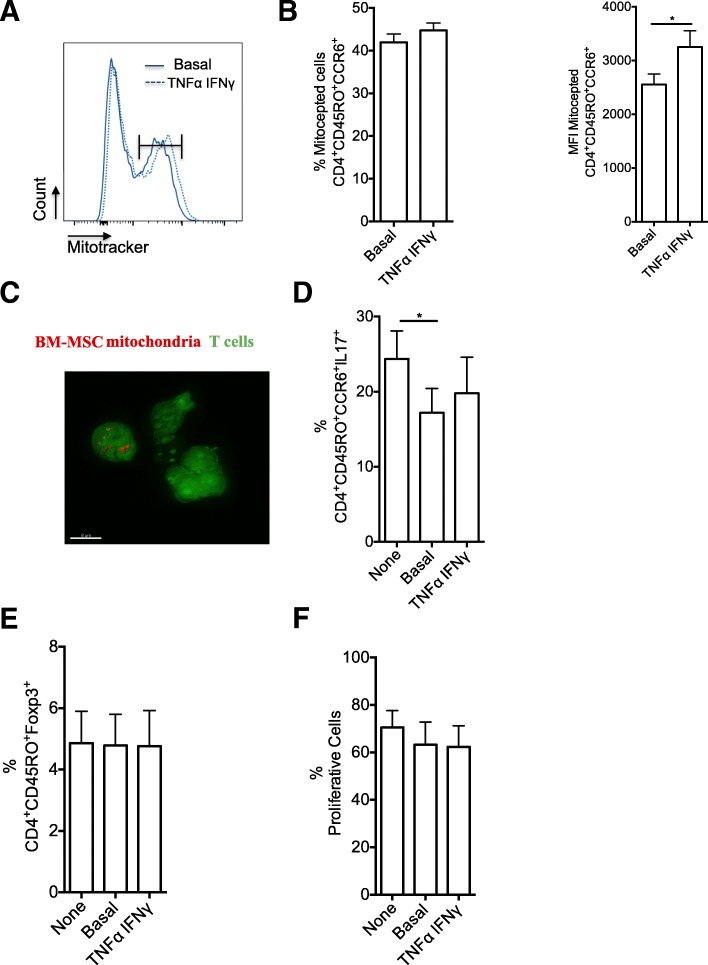
Artificial transfer of BM-MSC mitochondria in Th17 cells significantly reduces IL-17 production independently of TNF-α/IFN-γ activation. **a** Representative histogram showing MitoTracker-positive Th17 cells (flow cytometry) 24 h after artificial mitochondrial transfer using the MitoCeption protocol. Mitochondria were isolated from MitoTracker-labeled BM-MSCs pre-activated or not with TNF-α/IFN-γ. **b** Quantification of artificial mitochondrial transfer in Th17 cells as the percentage of MitoTracker-positive Th17 cells (left) or MFI (right). **c** Representative image of BM-MSC mitochondria after MitoCeption (ratio 0.025) in Th17 cells. **d** Quantification of IL-17 production by Th17 cells after 3 days of activation with beads coated with anti-CD3/CD28 antibodies following MitoCeption or not (None) of mitochondria isolated from BM-MSCs pre-activated or not (Basal) with TNF-α and IFN-γ. **e** FOXP3 expression quantification in Th17 cells after 3 days of activation with beads coated with anti-CD3/CD28 antibodies following MitoCeption or not (None) of mitochondria isolated from BM-MSCs pre-activated or not (Basal) with TNF-α and IFN-γ. **f** Proliferation of Th17 cells after 3 days of activation with beads coated with anti-/CD28 antibodies following MitoCeption or not (None) of mitochondria isolated from BM-MSCs pre-activated or not (Basal) with TNF-α and IFN-γ. Data are the mean ± SEM of at least 3 independent experiments using PBMCs from five donors and BM-MSCs from three donors and a ratio for MitoCeption of 1 BM-MSC:25 Th17 cells

To assess whether BM-MSC mitochondria have a role in the regulation of CD4^+^ Th17 cell effector functions, the previously described MitoCeption technique [17] was used to transfer mitochondria isolated from BM-MSCs to the target cells without the need of co-culture. Before mitochondria isolation, BM-MSCs were labeled with MitoTracker Deep Red and T cells with CellTracker (green). Flow cytometry analysis of T cells after MitoCeption revealed that almost 40% of T cells had incorporated the MitoTracker dye (Fig. 5a, b), a percentage similar to the one obtained after co-culture of BM-MSCs and T cells (Fig. 1b). 3D reconstruction of confocal microscopy images after MitoCeption highlighted the presence of BM-MSC mitochondria in T cells (Fig. 5c). Next, unlabeled BM-MSCs were used as a source of mitochondria for MitoCeption of Th17 cells. After MitoCeption, Th17 cells were activated with anti-CD3 and anti-CD28 antibody-coated beads for 3 days, and then their ability to produce IL-17 was assessed. The percentage of IL-17-producing cells (Fig. 5d) was significantly reduced among Th17 cells that underwent MitoCeption compared with controls (no MitoCeption). This indicates that BM-MSC mitochondria are sufficient to alter Th17 effector functions and suggests that mitochondrial transfer plays a role in MSC immunomodulation of Th17 cells. Finally, MitoCeption with mitochondria isolated from BM-MSC activated with pro-inflammatory cytokines (TNFα and IFNγ) did not affect the percentage of Th17 cells that incorporated mitochondria (Fig. 5a), but slightly increase the MFI (Fig. 5b). Moreover, mitochondria from activated BM-MSCs did not change the percentage of IL-17-producing Th17 cells after MitoCeption compared with mitochondria from non-activated BM-MSCs (Fig. 5d). The immunoregulatory effect mediated by mitochondrial transfer to Th17 cells was not associated with increased FOXP3 expression level (Fig. 5e). Then, to assess the effect of BM-MSC mitochondria transfer on T cell proliferation, MitoCepted T cells and control T cells were labeled with CellTrace violet. After 3 days of culture, the proliferation rate was comparable in control T cells and in T cells that underwent MitoCeption of mitochondria from non-activated and activated BM-MSCs (Fig. 5f).

### Transfer of mitochondria derived from healthy BM-MSCs and from RA-sMSCs

**Fig. 6:**
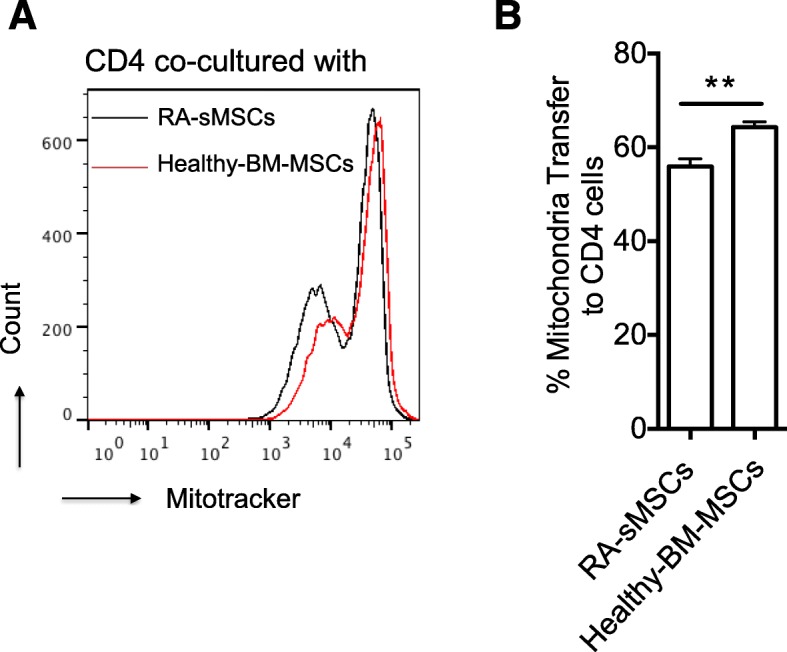
Mitochondrial transfer from RA-sMSCs to CD4^+^ T cells is less efficient than that from BM-MSCs. **a** Representative histogram of mitochondrial transfer from synovium-derived MSCs (patients with rheumatoid arthritis; RA-sMSCs) and from BM-MSCs (healthy donors) to CD4^+^ T cells. **b** Quantification of the percentage of mitochondrial transfer from RA-sMSCs and from BM-MSCs to CD4^+^ T cells. Data are the mean ± SEM of at least 3 independent experiments using PBMCs from three donors and BM-MSCs from three healthy donors or RA-MSCs from three patients with RA (PBMC to BM-MSC or RA-sMSC ratio = 1:25)

MSCs are the main stromal cells present in synovitis during active RA. Moreover, our previous findings showed that BM-derived and synovium-derived MSCs (sMSCs) share similar phenotypic properties and differentiation potential [21]. To determine whether these two cell types exhibit the same mitochondrial transfer potential and whether RA affects this process, phenotypically characterized RA-sMSCs (CD44^+^, CD29^+^, CD105^+^) and BM-MSCs from healthy donors were labeled with MitoTracker before co-culture with CD4^+^ T cells for 4 h. FACS analysis indicated that the mitochondrial transfer capacity of RA-sMSCs was significantly reduced compared with BM-MSCs (Fig. 6a, b). Indeed, 56% and 66% of CD4^+^CD45RO^+^ T cells were Mito+ after 4 h of co-culture with RA-sMSCs and BM-MSCs, respectively (*p* < 0.05) (Fig. 6b).

## Discussion

The present study shows that BM-MSC immunoregulatory effect on Th17 cells relies also on mitochondrial transfer. Mitochondrial transfer was observed already after 4 h of co-culture of BM-MSCs and T cells and did not require MSC activation by pre-incubation with pro-inflammatory cytokines. Moreover, after co-culture for 24 h, the MFI level was further increased, but not the percentage of T cells that acquired mitochondria. These findings suggest that a Th17 cell sub-population is resistant to mitochondrial transfer. Moreover, mitochondrial transfer was associated with increased oxygen consumption by Th17 cells that significantly impaired their capacity to produce IL-17. Additionally, the analysis of the Th17 cell phenotype after cell sorting according to the uptake or not of mitochondria showed a significant increase of Treg markers in Th17 cells that acquired mitochondria, suggesting that mitochondrial transfer from MSCs can promote the acquisition of an anti-inflammatory phenotype by pro-inflammatory Th17 cells. Finally, our study showed that mitochondrial transfer is impaired in sMSCs from patients with RA compared with BM-MSCs from healthy donors.

Intercellular communication allows the transfer of small molecules and also of intracellular structures, including components of the plasma membrane, lysosomes, endosomal vesicles, and mitochondria, unidirectionally and/or bidirectionally [15]. MSCs can transfer their mitochondria to target cells [23] to restore mitochondrial function in recipient cells [19, 20].

T cell activation and Th17 cell differentiation are associated with glycolysis increase, whereas mitochondrial alteration is associated with lipid oxidation [24]. T cell antigen receptor stimulation in the presence of inflammatory co-stimulation leads to activation of the PI3K/AKT/mTORC1 signaling pathway and promotes aerobic glycolysis and glutamine metabolism. This leads to increased lymphocyte proliferation and activation [25]. This metabolic phenotype is also associated with reduced expression levels of the Treg cell transcription factor FOXP3 and a decrease of Treg suppressive functions [26]. In response to T cell activation, a metabolic switch from OXPHOS to glycolysis occurs concomitantly with an increase in T cell size, indicating that activated T cells rely predominantly on glycolysis [27]. Mitochondrial lipid oxidation alters the electron flow through the succinate dehydrogenase complex, promoting the generation of reactive oxygen species and inflammation [28]. Furthermore, it has been shown that memory T cell development depends on mitochondrial fatty acid oxidation [29]. It has been proposed that high glycolytic activity favors Th17 cell differentiation, whereas mitochondrial lipid oxidation promotes Treg cell differentiation, suggesting that metabolic programming is required for T cell differentiation. Accordingly, the local nutrient conditions are critical for the regulation of the ratio between pathogenic Th17 and Treg cells. Remarkably, various cellular factors that are implicated in the activation of the glycolysis pathway in T cells (e.g., mTOR and HIF-1) directly target and activate the key molecules of the Th17 cell signature (the master transcription factor RORγt, the cytokines IL-17A and IL-17F, and IL-23 receptor) and induce the expression of various genes that support Th17 cell survival [30]. On the other hand, HIF1 promotes the proteosomal degradation of FOXP3, a Treg cell marker [31]. However, it is not well understood how these metabolic switches operate and how they are related to T cell plasticity.

MSCs can transfer mitochondria to T cells to compensate for mitochondrial energetic metabolic alterations. We thus hypothesized that mitochondrial transfer from MSCs to Th17 cells regulates their function and also chronic inflammation. Our data show that the rapid uptake of BM-MSC mitochondria by Th17 cells after co-culture has a significant impact on Th17 cell metabolism by increasing the maximal T cell respiration. In addition, IL-17 production by Th17 cells was decreased after artificial mitochondrial transfer, demonstrating that the suppressive effect associated with mitochondrial transfer in co-culture was driven only by mitochondria from MSCs. Importantly, this inhibitory activity is independent of BM-MSC pre-activation by incubation with IFN-γ and TNF-α. Altogether, these results suggest that MSCs exert an immunosuppressive activity on the Th17 cell subset via mitochondrial transfer that increases oxygen consumption and consequently induces OXPHOS. Additional studies are required to demonstrate the mechanism by which mitochondrial transfer modulates the phenotype and function of pathogenic Th17 cells, and the therapeutic relevance of this phenomenon in the context of autoimmune diseases.

## Conclusions

In RA, chronic synovitis is characterized by proliferation of synovial stromal cells and accumulation of monocytes and Th17 cells. Synovial cell ability to adapt their metabolism in response to the inflammatory microenvironment is involved in RA pathogenesis. In the RA joint, mitochondrial dysfunction is associated with oxidative stress, angiogenesis, pro-inflammatory cytokines, and activation of the NLRP3 inflammasome [32]. Furthermore, metabolic studies have shown that in the RA synovium, the nicotinamide adenine dinucleotide phosphate oxidase NOX2 is increased and cytochrome C oxidase is deficient [33]. In addition, alterations in mitochondrial function in response to hypoxia lead to a shift in synovial bioenergetics to glycolysis and induce pro-inflammatory mechanisms. In autoimmune diseases, such as systemic lupus erythematosus, MSCs influence T cell response by repressing autophagy following mitochondrial transfer [34]. Here, we found that mitochondrial transfer from RA-sMSCs to Th17 cells is significantly reduced compared with BM-MSCs from healthy donors. This defect could be associated with reduced MSC immunoregulatory effects on Th17 cells in RA. This suggests a putative new mechanism responsible for the persistence of active pathogenic Th17 cells in chronic arthritis. Additional studies are needed to demonstrate that the impaired mitochondrial transfer from sMSCs alters Th17 cell function in RA.

## Additional file

Additional file 1: **Figure S1.** Artificial transfer of mitochondria from BM-MSCs to Th17 cells using the “MitoCeption” protocol. Schematic representation of the different steps used for artificial mitochondrial transfer from BM-MSCs to Th17 cells. (PDF 583 kb)

## Data Availability

The datasets used and analyzed in this study are available from the corresponding author on reasonable request.

## Abbreviations

BM: Bone marrow
CTV: CellTrace Violet
FOXP3: Forkhead box protein P3
IDO: Indoleamine-2,3-dioxygenase
IFN: Interferon
IL: Interleukin
iNOS: Nitric oxide synthase
MSC: Mesenchymal stem cells
mtDNA: Mitochondrial DNA
OCR: Oxygen consumption rate
OXPHOS: Oxidative phosphorylation
PBMC: Peripheral blood mononuclear cells
PGE_2_: Prostaglandin 2
PHA: Phytohemagglutinin
RA: Rheumatoid arthritis
RORγ: Retinoid-related orphan receptor-γ
TNF: Tumor necrosis factor
TNT: Tunneling nanotubes
Treg: Regulatory T cell

## References

[1] Djouad F, Bouffi C, Ghannam S, Noel D, Jorgensen C (2009). Mesenchymal stem cells: innovative therapeutic tools for rheumatic diseases. Nat Rev Rheumatol.

[2] Yan L, Zheng D, Xu RH (2018). Critical role of tumor necrosis factor signaling in mesenchymal stem cell-based therapy for autoimmune and inflammatory diseases. Front Immunol.

[3] Luz-Crawford P, Jorgensen C, Djouad F (2017). Mesenchymal stem cells direct the immunological fate of macrophages. Results Probl Cell Differ.

[4] Su J, Chen X, Huang Y, Li W, Li J, Cao K, Cao G, Zhang L, Li F, Roberts AI (2014). Phylogenetic distinction of iNOS and IDO function in mesenchymal stem cell-mediated immunosuppression in mammalian species. Cell Death Differ.

[5] Augello A, Tasso R, Negrini SM, Amateis A, Indiveri F, Cancedda R, Pennesi G (2005). Bone marrow mesenchymal progenitor cells inhibit lymphocyte proliferation by activation of the programmed death 1 pathway. Eur J Immunol.

[6] Bouffi C, Bony C, Courties G, Jorgensen C, Noel D (2010). IL-6-dependent PGE2 secretion by mesenchymal stem cells inhibits local inflammation in experimental arthritis. PLoS One.

[7] English K, Ryan JM, Tobin L, Murphy MJ, Barry FP, Mahon BP (2009). Cell contact, prostaglandin E(2) and transforming growth factor beta 1 play non-redundant roles in human mesenchymal stem cell induction of CD4+CD25(high) forkhead box P3+ regulatory T cells. Clin Exp Immunol.

[8] Selmani Z, Naji A, Zidi I, Favier B, Gaiffe E, Obert L, Borg C, Saas P, Tiberghien P, Rouas-Freiss N (2008). Human leukocyte antigen-G5 secretion by human mesenchymal stem cells is required to suppress T lymphocyte and natural killer function and to induce CD4+CD25highFOXP3+ regulatory T cells. Stem Cells.

[9] Ghannam S, Pene J, Torcy-Moquet G, Jorgensen C, Yssel H (2010). Mesenchymal stem cells inhibit human Th17 cell differentiation and function and induce a T regulatory cell phenotype. J Immunol.

[10] Rozenberg A, Rezk A, Boivin MN, Darlington PJ, Nyirenda M, Li R, Jalili F, Winer R, Artsy EA, Uccelli A (2016). Human mesenchymal stem cells impact Th17 and Th1 responses through a prostaglandin E2 and myeloid-dependent mechanism. Stem Cells Transl Med.

[11] Luz-Crawford P, Noel D, Fernandez X, Khoury M, Figueroa F, Carrion F, Jorgensen C, Djouad F (2012). Mesenchymal stem cells repress Th17 molecular program through the PD-1 pathway. PLoS One.

[12] Djouad F, Plence P, Bony C, Tropel P, Apparailly F, Sany J, Noel D, Jorgensen C (2003). Immunosuppressive effect of mesenchymal stem cells favors tumor growth in allogeneic animals. Blood.

[13] Ren G, Zhao X, Zhang L, Zhang J, L'Huillier A, Ling W, Roberts AI, Le AD, Shi S, Shao C (2010). Inflammatory cytokine-induced intercellular adhesion molecule-1 and vascular cell adhesion molecule-1 in mesenchymal stem cells are critical for immunosuppression. J Immunol.

[14] English K, Barry FP, Field-Corbett CP, Mahon BP (2007). IFN-gamma and TNF-alpha differentially regulate immunomodulation by murine mesenchymal stem cells. Immunol Lett.

[15] Nzigou Mombo B, Gerbal-Chaloin S, Bokus A, Daujat-Chavanieu M, Jorgensen C, Hugnot JP, Vignais ML. MitoCeption: transferring isolated human MSC mitochondria to glioblastoma stem cells. J vis Exp. 2017;120:e55245. 10.3791/55245 PMC5409302 28287607

[16] Ahmad T, Mukherjee S, Pattnaik B, Kumar M, Singh S, Kumar M, Rehman R, Tiwari BK, Jha KA, Barhanpurkar AP (2014). Miro1 regulates intercellular mitochondrial transport & enhances mesenchymal stem cell rescue efficacy. EMBO J.

[17] Caicedo A, Fritz V, Brondello JM, Ayala M, Dennemont I, Abdellaoui N, de Fraipont F, Moisan A, Prouteau CA, Boukhaddaoui H (2015). MitoCeption as a new tool to assess the effects of mesenchymal stem/stromal cell mitochondria on cancer cell metabolism and function. Sci Rep.

[18] Babenko Valentina, Silachev Denis, Popkov Vasily, Zorova Ljubava, Pevzner Irina, Plotnikov Egor, Sukhikh Gennady, Zorov Dmitry (2018). Miro1 Enhances Mitochondria Transfer from Multipotent Mesenchymal Stem Cells (MMSC) to Neural Cells and Improves the Efficacy of Cell Recovery. Molecules.

[19] Paliwal S, Chaudhuri R, Agrawal A, Mohanty S (2018). Regenerative abilities of mesenchymal stem cells through mitochondrial transfer. J Biomed Sci.

[20] Paliwal S, Chaudhuri R, Agrawal A, Mohanty S (2018). Human tissue-specific MSCs demonstrate differential mitochondria transfer abilities that may determine their regenerative abilities. Stem Cell Res Ther.

[21] Djouad F, Bony C, Haupl T, Uze G, Lahlou N, Louis-Plence P, Apparailly F, Canovas F, Reme T, Sany J (2005). Transcriptional profiles discriminate bone marrow-derived and synovium-derived mesenchymal stem cells. Arthritis Res Ther.

[22] Lyons EA, Scheible MK, Sturk-Andreaggi K, Irwin JA, Just RS (2013). A high-throughput Sanger strategy for human mitochondrial genome sequencing. BMC Genomics.

[23] Wang J, Liu X, Qiu Y, Shi Y, Cai J, Wang B, Wei X, Ke Q, Sui X, Wang Y (2018). Cell adhesion-mediated mitochondria transfer contributes to mesenchymal stem cell-induced chemoresistance on T cell acute lymphoblastic leukemia cells. J Hematol Oncol.

[24] Frauwirth KA, Riley JL, Harris MH, Parry RV, Rathmell JC, Plas DR, Elstrom RL, June CH, Thompson CB (2002). The CD28 signaling pathway regulates glucose metabolism. Immunity.

[25] Jones RG, Pearce EJ (2017). MenTORing immunity: mTOR signaling in the development and function of tissue-resident immune cells. Immunity.

[26] Gerriets VA, Kishton RJ, Johnson MO, Cohen S, Siska PJ, Nichols AG, Warmoes MO, de Cubas AA, MacIver NJ, Locasale JW (2016). Foxp3 and Toll-like receptor signaling balance Treg cell anabolic metabolism for suppression. Nat Immunol.

[27] van der Windt GJ, Pearce EL (2012). Metabolic switching and fuel choice during T-cell differentiation and memory development. Immunol Rev.

[28] Mills EL, Kelly B, Logan A, Costa ASH, Varma M, Bryant CE, Tourlomousis P, Dabritz JHM, Gottlieb E, Latorre I (2016). Succinate dehydrogenase supports metabolic repurposing of mitochondria to drive inflammatory macrophages. Cell.

[29] Wang R, Green DR (2012). Metabolic checkpoints in activated T cells. Nat Immunol.

[30] Park MJ, Lee SH, Lee SH, Lee EJ, Kim EK, Choi JY, Cho ML (2015). IL-1 receptor blockade alleviates graft-versus-host disease through downregulation of an interleukin-1beta-dependent glycolytic pathway in Th17 cells. Mediat Inflamm.

[31] Shi LZ, Wang R, Huang G, Vogel P, Neale G, Green DR, Chi H (2011). HIF1alpha-dependent glycolytic pathway orchestrates a metabolic checkpoint for the differentiation of TH17 and Treg cells. J Exp Med.

[32] Biniecka M, Canavan M, McGarry T, Gao W, McCormick J, Cregan S, Gallagher L, Smith T, Phelan JJ, Ryan J (2016). Dysregulated bioenergetics: a key regulator of joint inflammation. Ann Rheum Dis.

[33] Cillero-Pastor B, Martin MA, Arenas J, Lopez-Armada MJ, Blanco FJ (2011). Effect of nitric oxide on mitochondrial activity of human synovial cells. BMC Musculoskelet Disord.

[34] Chen J, Wang Q, Feng X, Zhang Z, Geng L, Xu T, Wang D, Sun L (2016). Umbilical cord-derived mesenchymal stem cells suppress autophagy of T cells in patients with systemic lupus erythematosus via transfer of mitochondria. Stem Cells Int.

