# Supplementary figures and images for: Mesenchymal stem cell repression of Th17 cells is triggered by mitochondrial transfer
Source: Stem Cell Res Ther. 2019 Aug 1;10:232. doi: 10.1186/s13287-019-1307-9 (PMC6676586; doi:10.1186/s13287-019-1307-9)

Figure S1

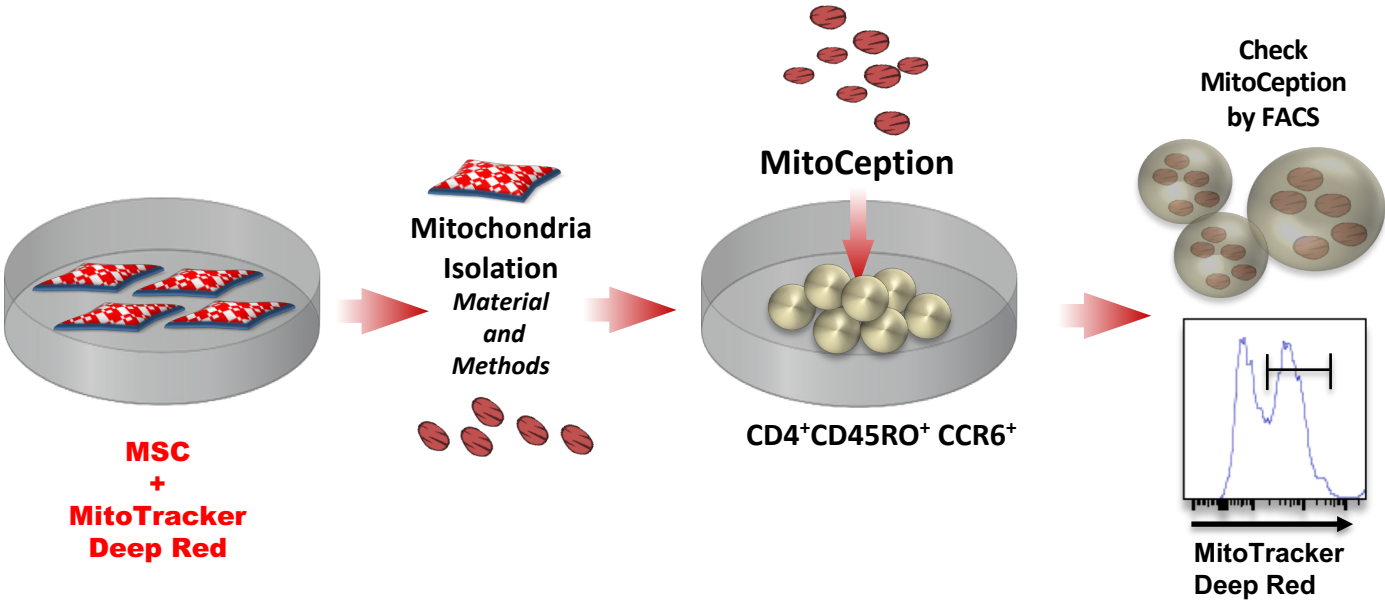

Supplement: Supplementary file 1 — Figure S1. Artificial transfer of mitochondria from BM-MSCs to Th17 cells using the “MitoCeption” protocol. Schematic representation of the different steps used for artificial mitochondrial transfer from BM-MSCs to Th17 cells. (PDF 583 kb) [file 13287_2019_1307_MOESM1_ESM.pdf]
